# Concentration of the Blood as a Cause of Convulsions

**Published:** 1888-03

**Authors:** 


### *CONCENTRATION OF THE BLOOD AS A CAUSE OF CONVULSIONS.*

The eminent Italian physiologist, Ivo Novi, has made a series of carefully-conducted experiments which prove that convulsions are frequently due to an increased specific gravity of the blood. The physiological and therapeutic effects of intravenous injections of chloride of sodium are already well known. Experiments made by earlier investigators have shown:

1. That the blood preserves its normal sp. gr. or amount of chloride of sodium by retarding or increasing excretion by the kidneys.

2. That a saturated solution of chloride of sodium will produce rigidity of muscles placed therein (Schiff).

3. That by increasing the amount of chloride sodium in the blood by intravenous injection, muscular spasms will be produced, which disappear when the sp. gr. of the blood is again reduced.

Novi has investigated the mode of production of these muscular contractions, endeavoring to determine whether they were the result of the formation of methæmoglobin, which produces the convulsions seen in asphyxia. He finds that methæmoglobin is not produced, but the concentration of the chlorides acts upon the tissues, purely by abstracting water, that is, by its hygroscopic power. He shows that the substance of the brain, especially the cortex, loses from five to six per cent. of its water.

The following is a summary of his conclusions:

1. The intravenous injection of a ten per cent. solution of chloride of sodium produces in all the higher mammals muscular spasms as soon as the sp. gr. of the blood becomes double the normal.

2. It does not produce this effect by converting the hæmoglobin into methæmoglobin, as in the case with the alkaline chlorates in the experiments of Marchand.

3. The peripheral nerves and muscles are not affected in these conditions. The central nervous system is affected, principally, by the abstraction of water from the brain, especially the gray substance of the cortex.

4. It is highly probable that the convulsions, which accompany analagous concentrations of the blood, as in Asiatic cholera, are due to the same cause.—*Lo Sperimentale.*

